# Prevalence of *H. pylori* among patients undergoing coronary angiography (The HP-DAPT prevalence study)

**DOI:** 10.1038/s41598-022-17034-0

**Published:** 2022-10-05

**Authors:** Karel Huard, Kevin Haddad, Yacine Saada, John Nguyen, David Banon, Alexis Matteau, Samer Mansour, Brian J. Potter

**Affiliations:** 1 Faculty of Medicine, Université de Montréal, Montreal, QC Canada; 2 Pharmacy Department, Centre Hospitalier de l’Université de Montréal (CHUM), Montreal, QC Canada; 3 Cardiovascular Centre, Department of Medicine, Centre Hospitalier de l’Université de Montréal (CHUM), Montreal, QC Canada; 4 Health Innovation and Evaluation Hub, Centre de Recherche du CHUM (CRCHUM), Montreal, QC Canada; 5 Cardiometabolic Division, Centre de Recherche du CHUM (CRCHUM), Montreal, QC Canada; 6 Carrefour de l’innovation et Évaluation en Santé (CIÉS), Centre de Recherche du CHUM (CRCHUM), Cardiology and Interventional Cardiology, CHUM, Pavillon S, S03-334, 850, Rue St-Denis, Montréal, QC H2X 0A9 Canada

**Keywords:** Cardiology, Interventional cardiology

## Abstract

*Helicobacter pylori* (*H. pylori*) screening and treatment is recommended for patients on chronic aspirin (ASA) therapy to reduce the risk of gastrointestinal bleeding. Coronary artery disease patients requiring combination antithrombotic therapy (dual antiplatelet therapy; DAPT, or dual pathway inhibition; DPI) are at an even higher risk of GI bleeding. Therefore, we aimed to evaluate the prevalence of *H. pylori* among patients referred for angiography and likely to receive DAPT or DPI. This single-center prospective observational study recruited patients undergoing coronary angiography and with the possibility of requiring DAPT or DPI. All included patients underwent *H. pylori* serology testing. Multivariable logistic regression was performed to determine predictors of seropositivity. 195 patients were included in the analysis. Mean age was 67 years, 50% had known prior CAD, and 49% underwent coronary intervention. *H. pylori* serology was positive in 36%. Chronic kidney disease (odds ratio [OR] 2.76; 95% confidence interval [CI] 1.24 to 6.15; *p* = 0.01) and chronic obstructive pulmonary disease (OR 2.52; 95% CI 1.14 to 5.55; *p* = 0.02) history were independent predictors of *H. pylori* seropositivity. Given the clinically significant prevalence of *H. pylori* seropositivity among patients referred for angiography, systematic screening strategies and eradication of *H. pylori* could significantly reduce the incidence of GI bleeding in patients requiring DAPT or DPI.

## Introduction

*Helicobacter pylori* (*H. pylori*) is both the most common chronic bacterial infection in humans and a significant cause of chronic gastritis, most peptic ulcers, and gastric adenocarcinoma and lymphoma^1,2^. A recent systematic review of *H. pylori* infection showed a prevalence of 37.1% in North America^3^ and it has been shown to affect up to 75% of individuals in specific populations^4^. Moreover, *H. pylori* infection is one of the most common cause of peptic ulcer disease^5^. Fortunately, eradication of *H. pylori* prevents recurrence and ulcer complications such as bleeding or perforation^6^.

Bleeding, most commonly gastro-intestinal (GI) bleeding^7^, is one of the most important adverse events associated with dual antiplatelet therapy (DAPT) and related antithrombotic regimens in patients with coronary artery disease (CAD). *H. pylori* infection has also been recognized as a risk factor for GI bleeding while on chronic daily ASA therapy^8^ and thienopyridine P2Y12-inhibitor antiplatelet therapy^9^. This risk is likely amplified with the use of novel, more potent P2Y12-inhibitor-based DAPT or when antiplatelet therapy must be combined with oral anticoagulation (dual-pathway inhibition; DPI). Some studies have also suggested that prolonged DAPT should be considered following acute coronary syndrome (ACS) for up to three years^10,11^, prolonging the period at increased of GI bleeding. The 2017 American College of Gastroenterology (ACG) guidelines recommend testing for *H. pylori* infection to reduce the risk of ulcer-related bleeding for those on chronic ASA therapy^12^. Despite this, such a strategy is still not common practice in those on antiplatelet monotherapy. Moreover, to our knowledge, systematic screening for *H. pylori* has not been prospectively evaluated for CAD patients receiving DAPT or DPI, who might derive even greater benefit from eradication therapy. In this study, we aimed to report the prevalence of positive *H. pylori* serology in an unselected population referred for coronary angiography and likely to required DAPT or DPI, as well as to determine clinical predictors of seropositivity.

## Methods

### Study design and setting

The HP-DAPT prevalence study was a single-center prospective observational study conducted at the *Centre hospitalier de l’Université de Montréal* (CHUM), a Canadian academic tertiary care center, between November 2018 and August 2019. Adult patients undergoing coronary angiography with the possibility of requiring DAPT or DPI afterwards were eligible for inclusion in this study, without restriction with regards to the acuity of the clinical presentation (unstable angina, NSTEMI, STEMI, and elective procedures included). Patients without the possibility of requiring DAPT or DPI post-procedure, such as those referred for diagnostic hemodynamic studies only, cardiac biopsy, or diagnostic angiography in anticipation of a surgical valve procedure, we excluded. Informed consent was obtained from all participants and all methods were carried out in accordance with the Declaration of Helsinki and its later amendments. The study protocol was approved by the institutional research ethics board of the CHUM.

### Data collection, clinical endpoints and H. pylori eradication

Clinical data was abstracted from the medical record and included demographics, medical history, laboratory values, baseline medications, pre-procedure investigations, clinical presentation, coronary procedures performed and discharge medications. Available medical records for data collection included both the electronic medical record from our center, as well as any paper documentation that would have patients transferred from referring hospitals. All clinical variables were prospectively identified and defined. Blood samples for *H. pylori* serology testing were drawn at the time of vascular access for coronary angiography. Specific anti–*H. pylori* IgGs were measured by use of a commercial ELISA (*H pylori* IgG ELISA, Bio-Rad Platelia™). Titers were defined as positive if optical density ratio was > 1.10 according to manufacturer’s instructions.

The primary endpoint of interest was the prevalence of *H. pylori* seropositivity. A key secondary objective was to determine clinical predictors of *H. pylori* seropositivity. Patients who were found to have positive serology were informed by phone. The patient’s primary caregiver was also informed by mail after obtaining the patient’s consent to communicate the results of the serology. Patients without a primary caregiver were offered eradication therapy by the research team. Eradication therapy, whether organized by the primary caregiver or the research team, was guided by a clinical decision aid (Table 1) developed by the investigators in accordance with published guidelines^13^ that took into account possible drug-drug interactions with the cardiac medications prescribed, such that the eradication regimen was adapted to the patient’s prescribed cardiac regimen. The preferred cardiac management of the treating team was therefore never altered to accommodate *H. pylori* eradication.

**Table 1:** *H. pylori* treatment algorithm in the presence of P2Y12-inhibitors or DOACs.

Agent used	Preferred regimen
Clopidogrel	Usual non-bismuth quadruple therapy PPI BID Amoxicillin 1000 mg BID Metronidazole 500 mg BID Clarithromycin 500 mg BID^1^ for 14 days
Prasugrel	
Ticagrelor	Bismuth quadruple therapy PPI BID Bismuth QID Metronidazole 500 mg TID or QID Tetracycline 500 mg QIDfor 14 days *Avoid clarithromycin* *If absolutely required, use bismuth subsalicylate with caution*^*2*^
DOACs	

^1^A reduction of Atorvastatin dose to 20 mg die is required with clarithromycin.

^2^Other bismuth preparations than bismuth subsalicylate should be used whenever possible.

*BID* twice daily; *DOACs* direct oral anticoagulants; *PPI* proton pump inhibitors.

### Statistical analysis

Statistical analyses were performed using the Statistical Package for Social Sciences (SPSS, IBM, Version 25). Normally distributed data are presented as means ± standard deviation and categorical data as counts with percentages of the total. Continuous variables were compared using a t-test when normally distributed and categorical data with the χ^2^ test. The difference in medians is estimated using the Hodges-Lehmann method where appropriate. Given the exploratory nature of the analysis, a two-tailed α of 0.05 was used for all analyses. Univariate predictors of positive *H. pylori* serology were identified using logistic regression. A multivariate logistic regression model was created including all univariate predictors with a *P* value < 0.05.

## Results

**Figure 1:**
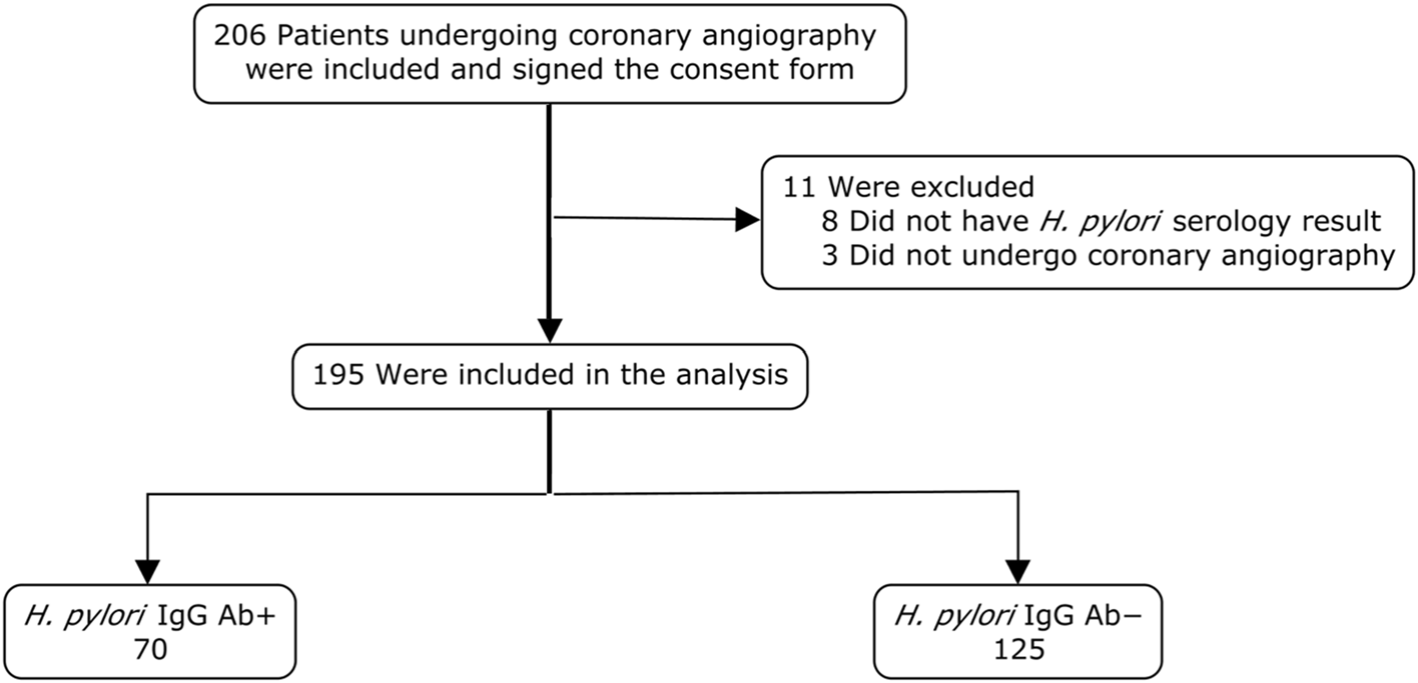
Patient flow diagram. *Ab* antibody.

From November 2018 to August 2019 a total of 206 patients provided consent, but serology was performed in only 195 (Fig. 1). Reasons for not performing serology included failure to obtain a blood sample because coronary angiography was ultimately not performed (3 patients) and laboratory mishandling of study samples (8 patients; *H. pylori* serology is no longer routinely performed for patients over 65 years of age at our center and 8 samples were not recognized as being study samples). Baseline patient characteristics are presented in Table 2. The mean age was 67.3 years, and 70.8% of patients were men. The vast majority of the cohort were Caucasian (90.3%). A history of hypertension was documented in 74% and 36% had diabetes. Pre-existing CAD was noted in 50% of patients, with 31% and 19% having had previous percutaneous coronary intervention (PCI) and coronary artery bypass grafting (CABG), respectively. A total of 69% had a history of atherosclerotic vascular disease.

**Table 2:** Baseline patient characteristics.

	Total (N = 195)	H Pylori + (N = 70)	H Pylori − (N = 125)	Odds ratio (95% CI)
Age (years) *mean* ± *SD*	67.3 $\pm$ 8.8	67.4 $\pm$ 8.8	67.2 $\pm$ 8.8	− 0.19 (− 2.79, 2.41) †
Male	138 (70.8)	54 (77.1)	84 (67.2)	0.87 (0.73, 1.04)
Caucasian Ethnicity	185 (94.9)	65 (92.9)	120 (96.0)	1.05 (0.97, 1.13)
Another Ethnicity	10 (5.1)	5 (7.1)	5 (4.0)	0.54 (0.14, 2.10)
**Hypertension**	144 (73.8)	56 (80.0)	88 (70.4)	0.88 (0.75, 1.04)
Resistant Hypertension^1^	5 (2.6)	3 (4.3)	2 (1.6)	0.37 (0.06, 2.18)
Diabetes	71 (36.4)	28 (40.0)	43 (34.4)	0.86 (0.59, 1.25)
Dyslipidemia	150 (76.9)	57 (81.4)	93 (74.4)	0.91 (0.79, 1.06)
**Vascular Disease**	134 (68.7)	50 (71.4)	84 (67.2)	0.95 (0.79, 1.15)
Coronary Artery Disease	98 (50.3)	37 (52.9)	61 (48.8)	0.92 (0.69, 1.23)
Previous PCI	61 (31.3)	19 (27.1)	42 (33.6)	1.24 (0.79, 1.95)
Previous CABG	37 (19.0)	13 (18.6)	24 (19.2)	1.03 (0.56, 1.90)
Peripheral Artery Disease	24 (12.3)	11 (15.7)	13 (10.4)	0.66 (0.31, 1.40)
CVA/TIA	15 (7.7)	6 (8.6)	9 (7.2)	0.84 (0.31, 2.26)
Heart Failure	31 (15.9)	13 (18.6)	18 (14.4)	0.78 (0.41, 1.49)
**Chronic Kidney Disease**^**2**^	33 (16.9)	18 (25.7)	15 (12.0)	0.47(0.25, 0.87)
Dialysis	1 (0.5)	1 (1.4)	0 (0.0)	–
Dyspepsia, Gastritis, or Esophagitis^3^	24 (12.3)	4 (5.7)	20 (16.0)	2.80 (1.00, 7.87)
GI Ulcer History^4^	7 (3.6)	2 (2.9)	5 (4.0)	1.40 (0.28, 7.03)
MALT lymphoma	0	0	0	–
Cirrhosis	5 (2.6)	2 (2.9)	3 (2.4)	0.84 (0.14, 4.91)
Alcohol Abuse History^5^	10 (5.1)	3 (4.3)	7 (5.6)	1.31 (0.35, 4.90)
Current NSAIDs Use	2 (1.0)	0 (0.0)	2 (1.6)	–
Bleeding History^6^	7 (3.6)	2 (2.9)	5 (4.0)	1.40 (0.28, 7.03)
COPD	34 (17.4)	18 (25.7)	16 (12.8)	0.50 (0.27, 0.91)
Atrial Fibrillation or Flutter	25 (12.8)	10 (14.3)	15 (12.0)	0.84 (0.40, 1.77)
Other ACO indications	7 (3.6)	2 (2.9)	5 (4.0)	1.40 (0.28, 7.03)

Values are counts and percentage of total unless otherwise stated.

^†^Age is presented as mean difference.

^1^Blood pressure > 140/90 despite three anti-hypertensive agents of different classes including a diuretic at maximum recommended doses.

^2^Chronic kidney disease was defined as eGFR less than 60 mL/min/1.73 m2.

^3^Clinical history of epigastric pain/burning or inflammation of the stomach or esophagus diagnosed with prior gastroscopy.

^4^History of gastric or duodenal ulcers diagnosed with gastroscopy.

^5^Current or past alcohol abuse (definition as per Canadian Centre on Substance Use and Addiction).

^6^History of bleeding diathesis (when present, type to specify).

*ACO* anticoagulation; *CABG* coronary artery bypass graft; *CI* confidence interval; *COPD* chronic obstructive pulmonary disease; *CVA* cerebrovascular accident; *GI* gastrointestinal; *MALT* mucosa-associated lymphoid tissue; *NSAIDs* nonsteroidal anti-inflammatory drugs; *PCI* percutaneous coronary intervention; *SD* standard deviation; *TIA* transient ischemic attack.

Angiographies were elective in half of patients, whereas NSTEMI was the leading cause of acute coronary syndrome presentation (Table 3). PCI was ultimately performed in 49% of patients. Slightly more than half of patients were discharged on two or more antithrombotic agents. There was no difference in the rates of antithrombotic agent class prescription at discharge between those with and without positive serology. Of note, *H. pylori* serology status was not known until after discharge in most cases.

**Table 3:** Pre-procedure and procedural clinical data.

	Total (N = 195)	H Pylori + (N = 70)	H Pylori − (N = 125)	Odds ratio (95% CI)
**Baseline laboratories**				
Hemoglobin (g/L) *mean* $\pm$ *SD*	134 $\pm$ 18	134 $\pm$ 18	135 $\pm$ 18	0.54 (− 4.88, 5.97) †
Platelets (× 10^9^/L) *median[IQR]*	222 [154,290]	215 [179,251]	213 [181,260]	1 (− 17, 19) ‡
Creatinine (μmol/L) *median[IQR]*	93 [44,142]	90 [73,122]	80 [64,96]	− 11 (− 18, − 3) ‡
INR *median[IQR]*	1.06 [0.84,1.28]	1.02 [0.98,1.10]	1.00 [0.96,1.10]	0.00 (− 0.04, 0.01) ‡
**Baseline medication**				
ASA	113 (57.9)	44 (62.9)	69 (55.2)	0.86 (0.68, 1.83)
P2Y12-inhibitors	30 (15.4)	8 (11.4)	22 (17.6)	1.53 (0.72, 3.25)
Vitamin K antagonists	2 (1.0)	1 (1.4)	1 (0.8)	0.57 (0.04, 8.96)
Direct oral anticoagulants	22 (11.3)	8 (11.4)	14 (11.2)	0.99 (0.44, 2.24)
Anticoagulant	24 (12.3)	9 (12.9)	15 (12.0)	0.94 (0.43, 2.04)
Proton pump inhibitors	77 (39.5)	22 (31.4)	55 (44.0)	1.37 (0.93, 2.04)
**Pre-procedure investigations**				
None performed	129 (66.2)	47 (67.1)	82 (65.6)	0.98 (0.79, 1.20)
Exercise stress test	22 (11.3)	7 (10.0)	15 (12.0)	1.20 (0.51, 2.80)
Nuclear stress test	38 (19.5)	15 (21.4)	23 (18.4)	0.86 (0.48, 1.54)
Stress Echo	8 (4.1)	3 (4.3)	5 (4.0)	0.93 (0.23, 3.79)
Negative test	3 (1.5)	1 (1.4)	2 (1.6)	1.12 (0.10, 12.13)
**Clinical presentation**				
Elective	96 (49.2)	36 (51.4)	60 (48.0)	0.93 (0.70, 1.25)
Unstable angina	21 (10.8)	5 (7.1)	16 (12.8)	1.79 (0.69, 4.68)
NSTEMI	68 (34.9)	23 (32.9)	45 (36.0)	1.10 (0.73, 1.65)
STEMI	10 (5.1)	6 (8.6)	4 (3.2)	0.37 (0.11, 1.28)
**Procedure**				
PCI	96 (49.2)	37 (52.9)	59 (47.2)	0.89 (0.67, 1.19)
Reference surgery	26 (13.3)	11 (15.7)	15 (12.0)	0.76 (0.37, 1.57)
**Discharge medication**				
ASA	166 (85.1)	60 (85.7)	106 (84.8)	0.99 (0.88, 1.11)
P2Y12-inhibitors	113 (57.9)	44 (62.9)	69 (55.2)	0.88 (0.69, 1.12)
Vitamin K antagonists	2 (1.0)	1 (1.4)	1 (0.8)	0.56 (0.04, 8.82)
Direct oral anticoagulants	27 (13.8)	10 (14.3)	17 (13.6)	0.95 (0.46, 1.96)
Anticoagulant	29 (14.9)	11 (15.7)	18 (14.4)	0.92 (0.46, 1.83)
Proton pump inhibitors	111 (56.9)	37 (52.8)	74 (59.2)	1.10 (0.85, 1.42)

Values are counts and percentage of total unless otherwise stated.

^†^Hemoglobin is presented as mean difference.

^‡^Platelets, Creatinine, and INR are presented as median difference.

*INR* international normalized ratio; *NSTEMI* non-ST-segment elevation myocardial infarction; *PCI* percutaneous coronary intervention; *STEMI* ST-segment elevation myocardial infarction.

**Figure 2:**
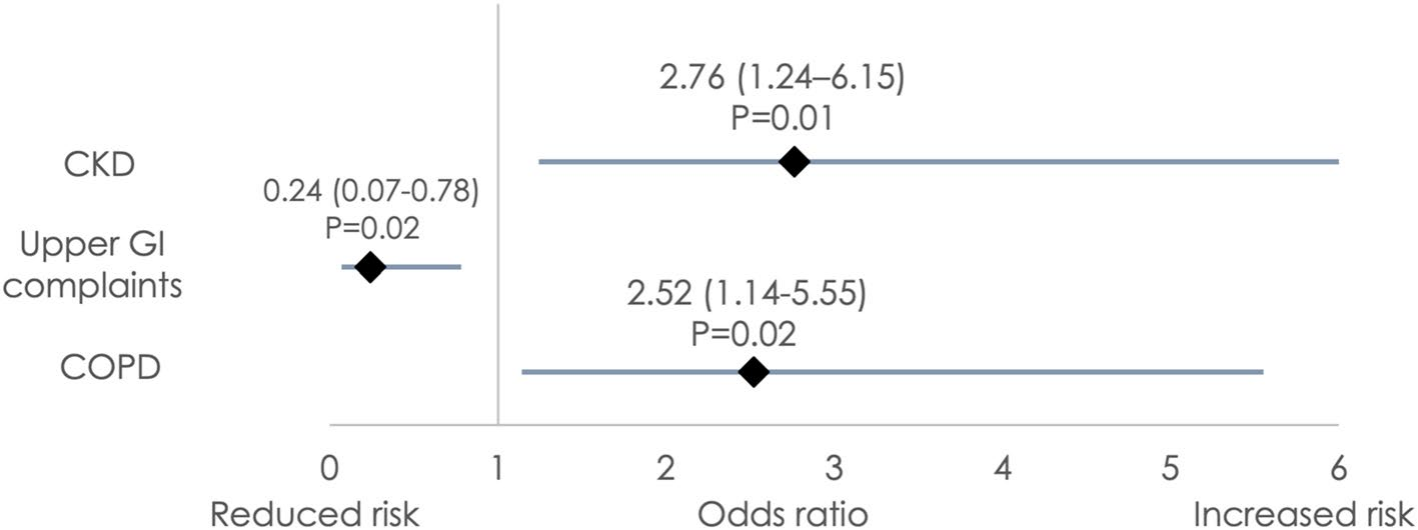
Predictors of *H. pylori* seropositivity based on multivariate analysis. *COPD* chronic obstructive pulmonary disease; *CKD* chronic kidney disease; *GI* gastrointestinal. A multivariate logistic regression model was created including all univariate predictors with a *P* value < 0.05. 95% confidence intervals are presented in this figure with the corresponding OR.

Independent predictors of *H. pylori* seropositivity (Fig. 2) included a history of chronic kidney disease defined as an estimated glomerular filtration rate (eGFR) less than 60 mL/min/1.73 m2 calculated with Chronic Kidney Disease Epidemiology Collaboration (CKD-EPI) equation (CKD; OR 2.76; 95% CI 1.24 to 6.15; *p* = 0.01) and chronic obstructive pulmonary disease (COPD; OR 2.52; 95% CI 1.14 to 5.55; *p* = 0.02). In contrast, patients with a history of dyspepsia, gastritis or esophagitis were less likely to be seropositive (OR 0.24; 95% CI 0.07 to 0.78; *p* = 0.02).

## Discussion

In this first Canadian study of *H. pylori* prevalence among CAD patients, the rate of positive *H. pylori* serology was 36% in a contemporary all-comers cohort of patients with the possibility of requiring DAPT or DPI, suggesting that a significant portion of the treated CAD population in Canada is at an increase, yet modifiable risk of GI bleeding.

While *H. Pylori* infection prevalence varies between and within countries^14^, our results are consistent with previously reported Canadian data (38% seropositivity in the general population)^3,15^. A large Canadian multicenter study also reported an *H. pylori* infection prevalence of 30% using histological assessment in patients with dyspepsia^16^. To the best of our knowledge, ours is the first study to specifically evaluate *H. pylori* prevalence in a Canadian CAD population and confirms that global estimates of *H. pylori* prevalence likely also apply to the CAD subpopulation.

Other studies of *H. pylori* prevalence in CAD populations have shown a wide range of positivity rates. In the United States, a series of 890 patients undergoing coronary angiography showed a 56% prevalence of *H. pylori* seropositivity^17^, whereas, in Europe, a German study showed a 44% seropositivity rate, but a United Kingdom analysis showed 79%^18,19^, reinforcing that knowledge of local seropositivity rates (and antibiotic resistances) should inform clinical decision-making.

In our cohort, three clinical characteristics were found to be independent predictors of seropositivity: CKD, COPD or a history of upper GI complaints (dyspepsia, gastritis or esophagitis), with the latter being protective. However, in the *H. pylori* literature, the strongest predictor of *H. pylori* infection is low socioeconomic status (SES) and poor living conditions early in life^20–22^. Factors such as overcrowding, lack of running water and poor hygiene have been linked to a higher rate of *H. pylori* infection, which tends to occur mainly during childhood^23^. It is possible that questioning patients on SES during childhood might have revealed additional risk factors. *H. pylori* seropositivity also increases with age^15^ and ethnicity is a risk factor with blacks and Hispanics having higher rates of infection^24^. It may therefore be that CKD and COPD are in fact surrogates for the combined effects of childhood SES and age at the time of angiography in our cohort and not necessarily themselves on the causal pathway of *H. pylori* seropositivity.

The association seen between a history of upper GI complaints and seronegativity is at first glance surprising. However, while some patients will remain seropositive following successful eradication therapy, others will seroconvert over time, particularly if there is a long interval between eradication therapy and repeat serology^25^. Therefore, it is possible that patients with a remote history of dyspepsia, gastritis or esophagitis had previously benefit from eradication therapy in the past and subsequently lost their anti-*H. pylori* antibodies over time, which could explain our results. Unfortunately, data on prior eradication therapy were not collected in our study. Alternatively, it is also possible that patients with a history of upper GI complaints with or without a history of *H. pylori* infection might have been less likely to be referred for angiography or might have been less inclined to consent for this study. Additionally, one must consider that many patients in our cohort were referred from other centers and nearly all were referred by a cardiologist. It is therefore possible that the non-cardiovascular medical history may not have been complete in all cases. As such, a documented history of GI complaints could simply be a marker of better overall care or, possibly, better SES. In addition, while we cannot evaluate this scenario with our dataset, it is conceivable that CAD is itself a strong predictor of seroposivity, such that a history of GI complaints may not have the same predictive impact as would be expected in the general population. The apparent protective effect of this variable observed here may therefore indeed be spurious due to a so-called index event bias (also known as collider stratification bias)^26,27^. For all of these reasons, the effect of a history of upper GI symptoms in this population should be interpreted with great caution.

Our results are also limited by the modest sample size of our exploratory study. Approximately 3300 diagnostic or interventional coronary procedures are performed annually in our center. A larger sample size could lead to both a refinement of the estimate of seropositivity and a clearer understanding of its predictors.

*H. pylori* serology is an inexpensive minimally invasive test with good sensitivity, but modest specificity for active infection (it cannot differentiate between current and prior infection). Studies have shown sensitivities ranging from 76 to 84% and specificities from 79 to 90%^28^. As such, treating *H. pylori* solely based on positive serology is controversial. Either a confirmatory urea breath test or stool antigen test is usually recommended before initiating treatment to confirm active infection. However, in the case of CAD patients receiving either DAPT or DPI, such confirmatory tests may not be immediately useful, as proton pump inhibitor (PPI) therapy increases the false negative rate of both the urea breath test^29,30^ and the stool antigen test^31^. Therefore, performing these confirmatory tests requires stopping PPI therapy for two weeks, which may not be considered optimal in patients on DAPT or DPI. In our study population, the majority patients were discharged on 2 or more antithrombotic agents and the majority were co-prescribed a PPI for gastric protection. Stopping PPI therapy could therefore increase the risk of bleeding for both patients with and without *H. pylori.* On the other hand, treating all patients with positive *H. pylori* serology would be expected to expose some false-positive (for active infection) patients to an unnecessary prolonged antibiotic treatment.

Moreover, typically recommended antibiotics against *H. pylori* infection are not always compatible certain P2Y12-inhibitors and direct oral anticoagulants (DOACs)^13^. Multiple regimens include macrolides, which are not compatible with either ticagrelor or any of the DOACs. Also, bismuth subsalicylate preparations have aspirin-like properties which can increase the risk of bleeding in the presence of other antithrombotic medication and should therefore be avoided or used with caution^32^. Therefore, screening for *H. pylori* and eradication as needed should ideally be performed prior to referral for elective coronary angiography whenever feasible. When not possible, such as in the case of an acute coronary syndrome, we believe that screening for *H. pylori* seropositivity should be considered at the time of admission, with discussion of the pros and cons of immediate eradication treatment according to the algorithm that we have proposed (Table 1).

## Conclusion

Given the clinically significant prevalence of positive *H. pylori* serology in this prospective Canadian cohort of patients referred for diagnostic coronary angiography, strategies of systematic screening and eradication of *H. pylori* could significantly reduce the incidence of GI bleeding in patients requiring DAPT or DPI. A prospective randomized clinical trial comparing either broad *H. pylori* screening or screening of patients with *H. pylori* risk factors compared to usual care is warranted.

## Data Availability

The datasets generated and analysed during the current study are available from the corresponding author on reasonable request.

## References

[1] Saleem N, Howden CW (2020). Update on the management of helicobacter pylori infection. Curr. Treat Opt. Gastroenterol..

[2] Crowe SE (2019). Helicobacter pylori Infection. N. Engl. J. Med..

[3] Hooi JKY, Lai WY, Ng WK, Suen MMY, Underwood FE, Tanyingoh D, Malfertheiner P, Graham DY, Wong VWS, Wu JCY, Chan FKL, Sung JJY, Kaplan GG, Ng SC (2017). Global prevalence of helicobacter pylori infection: Systematic review and meta-analysis. Gastroenterology.

[4] Cheung J, Goodman KJ, Girgis S (2014). Disease manifestations of Helicobacter pylori infection in Arctic Canada: Using epidemiology to address community concerns. BMJ Open.

[5] Lanas A, Chan FKL (2017). Peptic ulcer disease. Lancet.

[6] Wang AY, Peura DA (2011). The prevalence and incidence of Helicobacter pylori-associated peptic ulcer disease and upper gastrointestinal bleeding throughout the world. Gastrointest. Endosc. Clin. N. Am..

[7] Grove EL, Würtz SP, Jørgensen NR, Vestergaard P (2013). Gastrointestinal events with clopidogrel: A nationwide population-based cohort study. J. Gen. Intern. Med..

[8] Yeomans ND, Lanas AI, Talley NJ (2005). Prevalence and incidence of gastroduodenal ulcers during treatment with vascular protective doses of aspirin. Aliment Pharmacol. Ther..

[9] Abraham NS, Hlatky MA, Antman EM (2010). ACCF/ACG/AHA 2010 expert consensus document on the concomitant use of proton pump inhibitors and thienopyridines: A focused update of the ACCF/ACG/AHA 2008 expert consensus document on reducing the gastrointestinal risks of antiplatelet therapy and NSAID use. Circulation.

[10] Mauri L, Kereiakes DJ, Yeh RW (2015). Twelve or 30 months of dual antiplatelet therapy after drug-eluting stents. N. Engl. J. Med..

[11] Bonaca MO, Bhatt DL, Cohen M (2015). Long-term use of ticagrelor in patients with prior myocardial infarction. N. Engl. J. Med..

[12] Chey WD, Leontiadis GI, Howden CW, Moss SF (2017). ACG clinical guideline: Treatment of helicobacter pylori infection. Am. J. Gastroenterol..

[13] Fallone CA, Chiba N, van Zanten SV, Fischbach L, Gisbert JP, Hunt RH, Jones NL, Render C, Leontiadis GI, Moayyedi P, Marshall JK (2016). The toronto consensus for the treatment of helicobacter pylori infection in adults. Gastroenterology.

[14] Sjomina O, Pavlova J, Niv Y, Leja M (2018). Epidemiology of Helicobacter pylori infection. Helicobacter.

[15] Veldhuyzen van Zanten SJ, Pollak PT, Best LM, Bezanson GS, Marrie T (1994). Increasing prevalence of helicobacter pylori infection with age: Continuous risk of infection in adults rather than cohort effect. J. Infect. Dis..

[16] Thomson AB, Barkun AN, Armstrong D, Chiba N, White RJ, Daniels S, Escobedo S, Chakraborty B, Sinclair P, Van Zanten SJ (2003). The prevalence of clinically significant endoscopic findings in primary care patients with uninvestigated dyspepsia: the Canadian Adult Dyspepsia Empiric Treatment - Prompt Endoscopy (CADET-PE) study. Aliment Pharmacol. Ther..

[17] Zhu J, Nieto FJ, Horne BD, Anderson JL, Muhlestein JB, Epstein SE (2001). Prospective study of pathogen burden and risk of myocardial infarction or death. Circulation.

[18] Koenig W, Rothenbacher D, Hoffmeister A, Miller M, Bode G, Adler G, Hombach V, März W, Pepys MB, Brenner H (1999). Infection with Helicobacter pylori is not a major independent risk factor for stable coronary heart disease: Lack of a role of cytotoxin-associated protein A-positive strains and absence of a systemic inflammatory response. Circulation.

[19] Whincup P, Danesh J, Walker M, Lennon L, Thomson A, Appleby P, Hawkey C, Atherton J (2000). Prospective study of potentially virulent strains of Helicobacter pylori and coronary heart disease in middle-aged men. Circulation.

[20] Hunt RH, Sumanac K, Huang JQ (2001). Review article: should we kill or should we save Helicobacter pylori?. Aliment Pharmacol. Ther..

[21] Webb PM, Knight T, Greaves S, Wilson A, Newell DG, Elder J, Forman D (1994). Relation between infection with Helicobacter pylori and living conditions in childhood: Evidence for person to person transmission in early life. BMJ.

[22] Kivi M, Johansson AL, Reilly M, Tindberg Y (2005). Helicobacter pylori status in family members as risk factors for infection in children. Epidemiol. Infect..

[23] Mendall MA, Goggin PM, Molineaux N, Levy J, Toosy T, Strachan D, Northfield TC (1992). Childhood living conditions and Helicobacter pylori seropositivity in adult life. Lancet.

[24] Everhart JE, Kruszon-Moran D, Perez-Perez GI, Tralka TS, McQuillan G (2000). Seroprevalence and ethnic differences in Helicobacter pylori infection among adults in the United States. J. Infect. Dis..

[25] Cutler AF, Prasad VM, Santogade P (1998). Four-year trends in Helicobacter pylori IgG serology following successful eradication. Am. J. Med..

[26] Choi HK, Nguyen US, Niu J, Danaei G, Zhang Y (2014). Selection bias in rheumatic disease research. Nat. Rev. Rheumatol..

[27] Dahabreh IJ, Kent DM (2011). Index event bias as an explanation for the paradoxes of recurrence risk research. JAMA.

[28] Chey WD, Wong BC (2007). Practice parameters committee of the American college of gastroenterology. American college of gastroenterology guideline on the management of helicobacter pylori infection. Am. J. Gastroenterol..

[29] Laine L, Estrada R, Trujillo M, Knigge K, Fennerty MB (1998). Effect of proton-pump inhibitor therapy on diagnostic testing for Helicobacter pylori. Ann. Intern. Med..

[30] Bravo LE, Realpe JL, Campo C, Mera R, Correa P (1999). Effects of acid suppression and bismuth medications on the performance of diagnostic tests for Helicobacter pylori infection. Am. J. Gastroenterol..

[31] Gatta L, Vakil N, Ricci C, Osborn JF, Tampieri A, Perna F, Miglioli M, Vaira D (2004). Effect of proton pump inhibitors and antacid therapy on 13C urea breath tests and stool test for Helicobacter pylori infection. Am. J. Gastroenterol..

[32] Lexicomp. (n.d.). Bismuth subsalicylate: Drug information. *UpToDate*. Retrieved from 15 May 2021, from https://www.uptodate.com/contents/bismuth-subsalicylate-drug-information.

